# PD41 - Risk factors for side effects during venom immunotherapy in children with hymenoptera venom allergy

**DOI:** 10.1186/2045-7022-4-S1-P41

**Published:** 2014-02-28

**Authors:** Suleyman Tolga Yavuz, Umit Murat Sahiner, Betul Buyuktiryaki, Ebru Arik Yilmaz, Özlem Cavkaytar, Cansin Sackesen, Bulent E Sekerel, Ozge Uysal Soyer, Ayfer Tuncer

**Affiliations:** 1 Hacettepe University, School of Medicine, Pediatric Allergy and Asthma Unit, Ankara, Turkey

## Background

Since the side effects during Venom Immunotherapy (VIT) are associated with several risk factors, we aimed to evaluate the association of serum basal tryptase (sBT) levels and of other parameters with the frequency of local and/or systemic reactions during VIT in children.

## Method

Children who underwent conventional VIT due to established honeybee or wasp allergy and completed 1-year VIT were included in the study. Data were collected on sBT levels, age, sex, culprit insect, degree of preceding sting reaction, time between last preceding sting reaction and VIT, venom specific IgE concentration, total IgE levels, accompanying asthma and aeroallergen sensitization.

## Results

We enrolled 45 children with a mean ((± standart deviation) age of 10.0 ± 3.4 years. VIT with wasp venom was initiated in 39 patients (87%) and with honeybee venom in 6 patients (13%). Seventeen patients (37.8%) had encountered lokal or systemic side effects during VIT. Side effects were present in 41 out of 1448 injections (2.8%). There was no significant difference at sBT levels of children with (4.3 µg/L [3.7-6.3]) or without (4.2 µg/L [3.1-4.7]) side effects (p=0.303). Multivariate logistic regression analysis revealed presence of asthma (odds ratio; [95% confidence interval] (14.2 [2.0–123.5]; p=0.008) as a significant risk factor for side effects during VIT in children.

## Conclusion

Results of our study determined an association between accompanying asthma and side effects during VIT. Patients with asthma may need a particularly high degree of surveillance during VIT procedure.

